# Plant–soil feedback from eastern redcedar (*Juniperus virginiana*) inhibits the growth of grasses in encroaching range

**DOI:** 10.1002/ece3.9400

**Published:** 2022-10-17

**Authors:** Leland D. Bennion, David Ward

**Affiliations:** ^1^ Department of Biological Sciences Kent State University Kent Ohio USA

**Keywords:** allelopathy, eastern redcedar, *Juniperus virginiana*, plant–soil feedback, prairie, range expansion, soil community, woody encroachment

## Abstract

The encroachment of woody plants into grasslands is an ongoing global problem that is largely attributed to anthropogenic factors such as climate change and land management practices. Determining the mechanisms that drive successful encroachment is a critical step towards planning restoration and long‐term management strategies. Feedbacks between soil and aboveground communities can have a large influence on the fitness of plants and must be considered as potentially important drivers for woody encroachment. We conducted a plant–soil feedback experiment in a greenhouse between eastern redcedar *Juniperus virginiana* and four common North American prairie grass species. We assessed how soils that had been occupied by redcedar, a pervasive woody encroacher in the Great Plains of North America, affected the growth of *Andropogon gerardi*, *Schizachyrium scoparium*, *Bromus inermis*, and *Pascopyrum smithii* over time. We evaluated the effect of redcedar on grass performance by comparing the height and biomass of individuals that were grown in live or sterilized conspecific or redcedar soil. We found redcedar created a negative plant–soil feedback that limited the growth of the cool season grasses *B. inermis* and *P. smithii*, reducing their overall biomass by >60%. These effects were found in both live and sterilized redcedar soils. In live soils, some growth suppression can be attributed to the negative effects of soil microbes. The limitation of grass growth in sterile soils indicates redcedar may exude an allelochemical into the soil that limits grass growth. Our results demonstrate that plant–soil feedback created by redcedar inhibits the growth of certain grass species. By creating a plant–plant interaction that negatively affects competitors, redcedars increase the probability of seedling survival until they can grow to overtop their neighbors. These results indicate plant–soil feedback is a mechanism of native woody plant encroachment which could be important in many systems yet is understudied.

## Graphical abstract

The alteration of the near soil environment by plants, deemed plant–soil feedback, can have differential effects on conspecific and heterospecific plants and is a process that has been identified as an important contributor to the success of range‐expanding species. Using an experimental approach, we demonstrate that eastern redcedar (*Juniperus virginiana*), a widespread conifer that is encroaching into the grasslands of eastern North America, creates a plant–soil feedback that suppresses the growth of common prairie grasses. This suppressive effect could provide a crucial driver for the success of eastern redcedar establishment in highly competitive prairie plant communities.

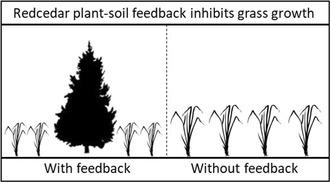

## 1 INTRODUCTION

Plants make species‐specific changes to the biotic and abiotic conditions of their near‐soil environment which can affect the fitness of future plants growing in that soil (Bever et al., 1997; Bezemer et al., 2006; Gundale & Kardol, 2021). This phenomenon, deemed plant–soil feedback, can have a large influence on competitive interactions, community composition and function (Crawford et al., 2019; Lekberg et al., 2018; van der Putten et al., 2013). The strength and direction of a feedback is the product of several interacting mechanisms including soil nutrient availability, the presence of pathogenic natural enemies and beneficial mutualists, and the effects of secondary chemicals (i.e., allelochemicals) exuded from plants (Bennett & Klironomos, 2019).

Plant–soil feedback is a well‐documented mechanism that can favor the fitness of range‐expanding and invasive species in plant communities (Aldorfová et al., 2020; Kulmatiski & Kardol, 2008). A typical experimental approach to determine if the soil microbial community is driving plant–soil feedbacks is to compare plant growth in soils with live microbial communities with soils that have had their microbial communities sterilized with heat or fungicides (Gundale et al., 2019; Kulmatiski & Kardol, 2008). Soil with live microbial communities can show a feedback not found in sterile communities due to the presence of beneficial or deleterious microbes (e.g., Cortois et al., 2016). Greenhouse feedback‐experiments typically have a training phase, during which soil is conditioned by the growth of a species of interest and a phytometer phase, where plants are grown in the training soil to evaluate whether a feedback affects their growth. A positive feedback occurs when the fitness of subsequent conspecific (plants of the same species) or heterospecific (plants of a different species) plants benefit from growing in soil altered (conditioned) by a given species. Conversely, a negative feedback describes a reduction in fitness when growing in conditioned soil (Kulmatiski et al., 2008). Plant–soil feedback could favor an encroaching species if it benefits the encroacher (intra‐ or interspecific positive feedback) or inhibits competitors (interspecific negative feedback) or both (Aldorfová et al., 2020; Bever et al., 1997).

Woody plant encroachment into grasslands is a global phenomenon that alters ecosystem function (Eldridge et al., 2011; Naito & Cairns, 2011). The conversion of grasslands to woodlands can decrease biodiversity, change ecosystem structure and function, reduce productivity for livestock, alter water resource availability, and change the carbon balance (Acharya et al., 2018; Anadón et al., 2014; Barger et al., 2011; Ratajczak et al., 2012). Encroachment can yield beneficial results such as marketable timber and nontimber products, creation of wildlife habitat, or a net gain in sequestered carbon (Archer, 2009; Stafford et al., 2017). Managing for encroaching species is difficult because the factors that are influential in range expansion differ between study species and systems (Tomiolo & Ward, 2018). Fire suppression and livestock grazing are land‐management practices frequently cited as the primary drivers of woody plant encroachment (Briggs et al., 2005; Van Auken, 2009). Regularly occurring fire can reduce the chances of successful establishment of trees in grasslands. Livestock often preferentially graze on grasses and herbaceous species and physically disturb the soil which can facilitate the encroachment of woody species (Archer et al., 2017). The global trend of climate change, specifically increased temperature, nutrient deposition and elevated CO_2_ levels, may also explain continental‐scale patterns of woody species expansion (Devine et al., 2017). An additional factor that may facilitate encroachment is plant–soil feedback, a mechanism that can promote the establishment of woody species and reinforce the dominance of a woody state (Peters et al., 2020).

In North America, woody encroachment is occurring in the deserts and rangelands of the west, the savannas of the south, and the grasslands of the Great Plains region (Ratajczak et al., 2012; Van Auken, 2000). Tree cover in rangelands of the western United States has increased by as much as 50% in the last 30 years, resulting in ~$5 billion in lost revenue (Morford et al., 2022). Encroachment in the Great Plains region of the United States is particularly concerning, with invading woody shrubs (e.g., *Cornus drummondii*) and trees (e.g., *Juniperus virginiana*) replacing grassland plant communities at a rate of up to 1.7% per year (Barger et al., 2011).

Understanding how successful woody encroachers establish and spread is critical to being able to manage them effectively and efficiently. It is of particular importance to understand mechanisms that provide an advantage to species in their expanded range and to quantify the strength of that advantage. This paper explores plant–soil feedback as a potential mechanism that has facilitated the movement of eastern redcedar (*J. virginiana*) from its historical range into the prairies of the Great Plains and into disturbed areas within their current ranges. Eastern redcedar (hereafter redcedar) is the most common, widely distributed conifer native to eastern North America (Fowells, 1965; Ward, 2020).

Redcedar tolerates a wide variety of climatic conditions including temperature extremes and drought. Redcedar is considered a long‐lived, early seral species and can be dominant in a forest or woodland habitat until later seral species establish (Lawson, 1990; Briggs et al., 2002). Historically, populations persisted where there was reduced threat of fire, such as on rocky outcrops or barrens (Briggs et al., 2002; Guyette et al., 2002). Several mechanisms have been proposed explaining why redcedar is a successful encroacher. In tallgrass prairies there is strong evidence for the interaction of intense livestock grazing and land management practices that have greatly extended fire‐return intervals beyond their pre‐European settlement levels as being determinants of redcedar expansion (Bielski et al., 2021; Briggs et al., 2005; Fogarty et al., 2021). There is also some evidence that the C_3_ photosynthetic pathway may provide an advantage to redcedar trees under elevated CO_2_ conditions over many of the warm‐season C_4_ grasses that co‐occur in its range (Huntley & Baxter, 2013; Iverson et al., 2008).

We conducted a fully crossed greenhouse experiment between redcedar and four common North American prairie grasses (*Andropogon gerardi*, *Schizachyrium scoparium*, *Bromus inermis*, *Pascopyrum smithii*). The analyses presented here evaluate whether redcedar creates plant–soil feedback with those grass species and determines the strength and direction of that feedback relative to feedbacks in conspecific soil. If plant–soil feedbacks are a mechanism that help redcedars following encroachment into prairies, we hypothesize we would observe the following outcomes: (a) grass growth in redcedar soils would be reduced more when compared to growth in intraspecific soils; (b) grass growth in live redcedar soil would be reduced when compared to sterile redcedar soil.

## 2 MATERIALS AND METHODS

### 2.1 Study species

We selected four common perennial grass species to be phytometers of soil conditioned by eastern redcedar. We selected two C_3_ and two C_4_ grasses for this experiment because both photosynthetic pathways are common in North America and frequently co‐occur, although they partition dominance along a gradient of temperature at the continental scale with C_3_ grasses predominating in the cooler north and C_4_ grasses in the warmer south (Still et al., 2003; Teeri & Stowe, 1976). *Andropogon gerardi* (big bluestem) and *Schizachyrium scoparium* (little bluestem) are common, native warm‐season C_4_ bunchgrasses with overlapping ranges in tall‐ or mixed‐grass prairies (Wang et al., 2013; Weaver, 1954). *Pascopyrum smithii* (western wheatgrass) is a common, native cool‐season C_3_ rhizomatous grass occurring in mixed‐grass prairies (Dong et al., 2014). *Bromus inermis* (smooth brome) is a common, Eurasian cool‐season C_3_ rhizomatous grass that has rapidly spread across North American grasslands since its introduction in the late 1800s (Vogel, 2004). *B. inermis* occurs in all contiguous states of the United States. All four grass species can co‐occur with each other and with redcedar in portions of their range (Burns, 1990; Weaver, 1942).

### 2.2 Phase I: Training phase

In the training phase of the experiment individuals were grown in potting mix to condition (or train) soils for use in the feedback phase. In February 2020 four shallow trays were filled with sterilized sand. Sand was steam sterilized in a pressurized autoclave at 121°C for ~60 min, cooled and then sterilized for an additional cycle (e.g., Crawford & Knight, 2017). Each tray was sown with an unsterilized monoculture of *A. gerardi*, *S. scoparium, B. inermis*, or *P. smithii* seeds. All seeds were purchased from OPN Seed, Ohio, USA. In early March 2020, 30 seedlings (mean grass height ~ 5 cm) of each species were transplanted into 5.6‐L pots of ProMix Ultimate potting mix (120 pots total). Plants were grown in a greenhouse and received auxiliary lighting using 1000 W high pressure sodium bulbs from 5:00–8:00 PM nightly to promote growth. We deemed the timespan from seedling to mature flowering grass was sufficient to train soils. Ten randomly selected pots of each grass species were harvested in mid‐June following ~16 weeks of growth. In addition, we randomly selected 10 pots from a pool of ~18‐month‐old redcedars that had been growing in 5.6 L pots of the same common potting soil in the same greenhouse for the previous 10 months. Prior to growth in our greenhouse, these redcedar seedlings were greenhouse‐raised at Pinelands Nursery, New Jersey, USA. Due to their slower overall growth rate, we determined that approximately 1 year of growth was sufficient to train soils for this experiment. Grass and tree samples were clipped at the root collar and aboveground biomass was dried in a 65°C oven and weighed. Training soils were separated from root materials manually by running material through a 2 mm sieve. Half of the soil (>2 L) collected from each sample was set aside for sterilization in an autoclave. Each pot was processed individually, and all materials used in processing were sterilized with an alcohol solution in‐between each sample. This procedure was established to prevent the transfer of soil particles and microbes between samples.

### 2.3 Phase II: Phytometer phase

**FIGURE 1:**
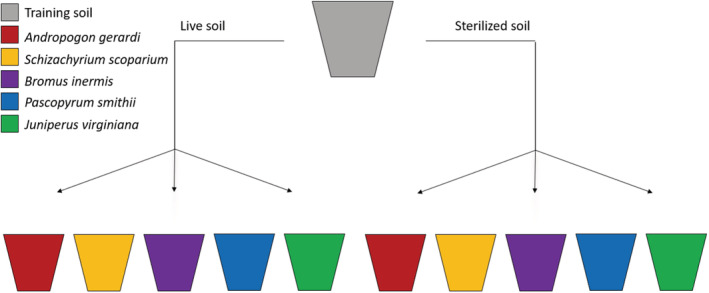
Illustration showing how soil from each training pot was distributed to 10 new pots for the phytometer phase. There were 50 total training sample pots, 10 from each study species. The gray pot represents one of the 50 training pots. The remaining pots are colored according to the phytometer that was grown in the soil conditioned by a given species in the training phase.

In June 2020, we germinated seeds of the same four grass species following the procedure outlined above. Eastern redcedar were purchased from Pinewoods nursery, New Jersey. Individual grass and redcedar seedlings were transferred into 2.8‐L pots that contained *home* or *redcedar* soils that were either *live* or *sterilized*. They were planted in pots using the following method: We added 1.3 L of sterilized sand, then 0.4 L of conditioned training soil from one of the five above‐mentioned species, followed by a 0.3 L cap of sterilized sand (Figure S1). We used a full‐factorial design with 10 replicates of each phytometer‐ and conditioned‐soil combination, resulting in a total of 500 experimental pots (Figure 1). Grasses were grown in controlled greenhouse conditions for 96 days. Eastern redcedars were allowed to grow for 13 months due to their slower growth rate. The maximum height of each plant was measured twice a week for the duration of the experiment. Pots were randomized at the beginning of the experiment and rotated every 4 weeks. All pots were watered ad libitum. At the end of the experiment the above‐ and belowground biomass in each sample were separated by cutting at the root collar. Belowground samples were rinsed thoroughly using a series of screens to prevent loss of fine roots. Above‐ and belowground samples were dried in an oven at 65°C for over 48 h prior to weighing.

### 2.4 Statistical analysis

Height data were recorded at regular intervals over the course of the experiment to aid in determining when plant–soil feedbacks occurred and to assess their strength and direction. The rate of plant growth is variable over time, which means nonlinear models will generally perform better than linear models at capturing how height changes over time. We chose to use generalized additive models (GAMs) to evaluate grass growth over time. GAMs are similar to generalized linear models except that they replace linear covariates with local smoothing functions that enable modeling of nonlinear processes (Hastie & Tibshirani, 1986). To help us understand the overall effect and timing of plant–soil feedbacks on the four phytometers, we built GAMs of the height data of each treatment group over time using the *mgcv* package (v1.8‐34; Wood, 2011) in R. The following is a simplification of the generalized additive model (GAM) formula that was used for each group of phytometers (Yee & Mitchell, 1991). 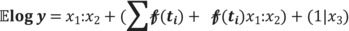 The formula relates the expected value (E) log_10_‐transformed height (logy) as a function of the interaction between the factors conditioning species ($x_{1}$) and sterilization status ($x_{2}$), the sum (∑) of smoothing 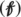 variables time ($t_{i}$) and time given each level of the interaction of the two factors 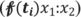, and a random intercept $\left(1|x_{3}\right)$ using the unique ID for each pot in the phytometer phase of the experiment. The random intercept was selected to account for repeated measures on each phytometer (Pedersen et al., 2019). The models used the Gaussian family and identity link function. Model selection was done by comparing the AIC for candidate models. We found this model formulation to explain the most variance while retaining only the variables that contribute to explanatory power of the model. We plotted the output of these generalized additive models (GAMs) using the *tidymv* R package to visualize and facilitate comparison of plant height over time under different treatments (Coretta, 2022). Post hoc comparisons were done using the *emmeans* package (v1.7.1‐1; Lenth, 2021). For each phytometer species, the mean estimated height was contrasted between each treatment group. Significance was determined using a Tukey post hoc comparison adjustment for a family of 10 estimates at the *p* < .05 level.

We assessed how the aboveground, belowground, and overall biomass differed between treatments, splitting the dataset into observations from each phytometer species. We ran a mixed‐effects model (GLMM) relating biomass (transformed to the log_10_ scale) as a function of the conditioning species, the sterilization status of the soil, and the interaction between the two. The pot ID number of the conditioned training soil was used as a random intercept with a fixed mean. Conditioned soils came from individual pots in the training stage that may differ in their abiotic and biotic features, so we chose to use mixed‐effects models to account for the variance in the strength of feedback due to these differences. A random effect was determined to be meaningful if the variance differed from zero, indicating individual pots from the training stage differed in their effect on the feedback. If the random effect was not meaningful, we ran the same formula as a generalized linear model (GLM). For GLMMs or GLMs of aboveground, belowground, and overall biomass data, the most parsimonious model was selected through comparison of AIC between full and reduced models. The type of model, whether an interaction term was used, and the *R* ^2^ value for each model is indicated (Table 1). To determine if any of the simple main effects were significant, we ran the same formula as an ANOVA using the linear model to calculate degrees of freedom and sum of squares error. We were particularly interested in comparing the effects of live and sterilized eastern redcedar soil to live and sterile home soils for each phytometer species. To elucidate this relationship for each phytometer species, we performed post hoc pairwise comparisons to obtain the estimated marginal means (also called least‐squares means) using the *emmeans* package (Lenth, 2021).

**TABLE 1:** The model type and *R* ^2^ value for each biomass type (shoot, root, or Total) and phytometer *Andropogon gerardi* (ANGE), *Schizachyrium scoparium* (SCSC), *Bromus inermis* (BRIN), and *Pascopyrum smithii* (PASM). Model types are mixed effects (M) or linear (L) and either contain an interaction term (I) between conditioning soil type and sterilization status or do not include the interaction term (no I). Asterisks (*) denote models that have significant main effects. Adjusted *R* ^2^ (adj) quantifies the explained variance of fixed effects in linear models. Conditional *R* ^2^ (cond) quantifies the variance described by fixed and random effects in mixed models. See methods section for detailed model description.

Phytometer	Biomass	Model type	*R* ^2^ (type)
ANGE	Shoot	M, I, *	.81 (cond)
ANGE	Root	M, I, *	.73 (cond)
ANGE	Total	M, I, *	.76 (cond)
SCSC	Shoot	L, I	.01 (adj)
SCSC	Root	L, I	.01 (adj)
SCSC	Total	L, I	.02 (adj)
BRIN	Shoot	L, I, *	.63 (adj)
BRIN	Root	M, I, *	.51 (cond)
BRIN	Total	L, I, *	.54 (adj)
PASM	Shoot	L, I, *	.53 (adj)
PASM	Root	M, no I, *	.66 (cond)
PASM	Total	M, no I, *	.64 (cond)

**FIGURE 2:**
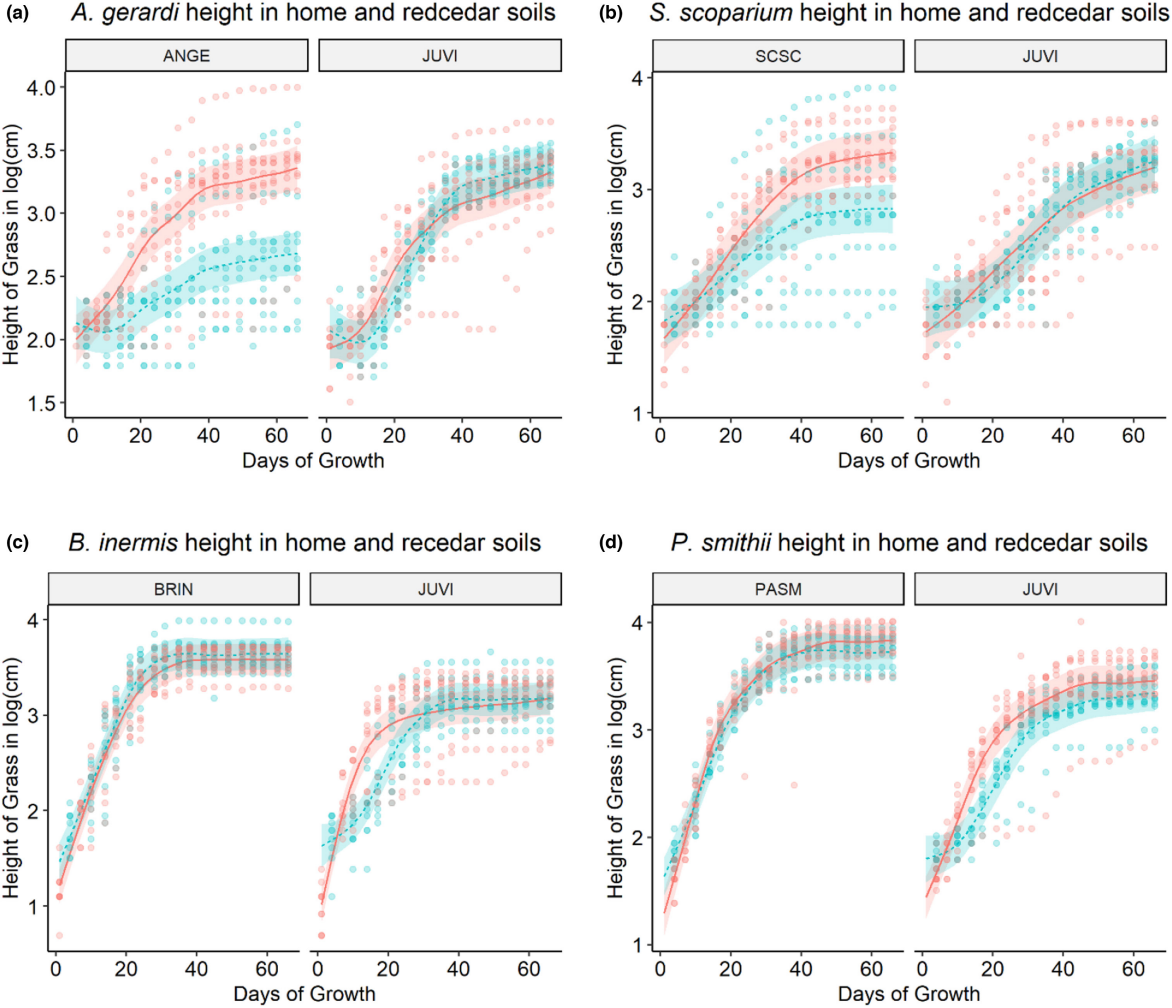
Plot showing modeled grass heights (mean line and 95% confidence intervals) and raw data (points) for the phytometers (a) *Andropogon gerardi*, (b) *Schizachyrium scoparium*, (c) *Bromus inermis*, and (d) *Pascopyrum smithii* grown in their home or *Juniperus virginiana* soils. The shaded areas illustrate 95% confidence intervals. Modeled means and confidence intervals are derived from the output of generalized additive models of log_10_(height) as a function of the interaction between the factors *soil sterilization status* and *conditioning soil type* and the smoothing variables *days of growth*, *days of growth given the interaction of treatment factors*, and the random intercept of *pot ID* for each plant. Grasses grown in live or sterile soils are indicated by red or blue coloration, respectively. Species names within each subfigure are abbreviated as follows: *A. gerardi* (ANGE), *S. scoparium* (SCSC), *B. inermis* (BRIN), *P. smithii* (PASM), and *J. virginiana* (JUVI).

We visualized differences in phytometer biomass between live and sterile home and redcedar soils using effects plots that were derived from the linear model fit for each set of contrasts (Ho et al., 2019; Wilschut & Van Kleunen, 2021). These plots illustrate simple mean differences between contrasts of interest with 95% confidence intervals using the sample data. The second part of these plots shows the modeled means and 95% confidence intervals paired with raw data points (Figure 2).

## 3 RESULTS

In general, soils conditioned by *Juniperus virginiana* (redcedar) suppressed the C_3_ grasses *Pascopyrum smithii* and *Bromus inermis* relative to growth in their home soils (Table 2 and Table S1). The C_4_ grasses *Andropogon gerardi* and *Schizachyrium scoparium* showed mixed feedbacks in soil conditioned by redcedar when compared to the height and biomass of plants grown in their home soils (Table 2 and Table S1).

**TABLE 2:** The mean estimate, variance, and confidence intervals of effects on shoot biomass for contrasting interactions of each home and redcedar (JUVI) and soil sterilization status. Phytometers and conditioned soil types are abbreviated as follows: *Andropogon gerardi* (ANGE), *Bromus inermis* (BRIN), *Pascopyrum smithii* (PASM), *Schizachyrium scoparium* (SCSC), and *Juniperus virginiana* (JUVI). Soils are either live (L) or sterile (S).

Phytometer	Contrasts	Estimate	SE	Df	Lower CL	Upper CL	*t* ratio	*p*
ANGE	JUVI L–ANGE L	1.662	0.324	25.382	0.771	2.554	5.126	<.001
	ANGE S–ANGE L	1.279	0.193	18	0.734	1.823	6.634	<.001
	JUVI L–ANGE S	0.384	0.324	25.382	−0.508	1.275	1.183	.643
	JUVI S–ANGE L	1.509	0.324	25.382	0.618	2.4	4.652	<.001
	JUVI S–ANGE S	0.23	0.324	25.382	−0.661	1.121	0.709	.892
	JUVI S–JUVI L	−0.154	0.193	18	−0.698	0.391	−0.797	.855
SCSC	JUVI L–SCSC L	0.405	0.773	36	−1.677	2.487	0.524	.953
	JUVI L–JUVI S	1.049	0.773	36	−1.033	3.132	1.357	.534
	JUVI L–SCSC S	−0.332	0.773	36	−2.415	1.75	−0.43	.973
	JUVI S–SCSC L	−0.644	0.773	36	−2.726	1.438	−0.833	.838
	SCSC L–SCSC S	−0.737	0.773	36	−2.82	1.345	−0.954	.776
	JUVI S–SCSC S	−1.382	0.773	36	−3.464	0.701	−1.787	.296
BRIN	JUVI L–BRIN L	−0.987	0.155	36	−1.405	−0.57	−6.373	<.001
	BRIN S–BRIN L	0.096	0.155	36	−0.322	0.513	0.618	.926
	JUVI L–BRIN S	−1.083	0.155	36	−1.5	−0.666	−6.991	<.001
	JUVI S–BRIN L	−0.708	0.155	36	−1.125	−0.29	−4.567	<.001
	JUVI S–JUVI L	0.28	0.155	36	−0.137	0.697	1.806	.287
	JUVI S–BRIN S	−0.803	0.155	36	−1.221	−0.386	−5.185	<.001
PASM	JUVI L–PASM L	−0.925	0.198	36	−1.46	−0.391	−4.663	<.001
	JUVI L–JUVI S	−0.301	0.198	36	−0.835	0.233	−1.517	.438
	JUVI L–PASM S	−1.206	0.198	36	−1.741	−0.672	−6.079	<.001
	JUVI S–PASM L	−0.624	0.198	36	−1.159	−0.09	−3.145	.017
	PASM L–PASM S	−0.281	0.198	36	−0.816	0.253	−1.416	.498
	JUVI S–PASM S	−0.905	0.198	36	−1.44	−0.371	−4.562	<.001

### 3.1 Plant height

Comparisons between the estimated mean height of each phytometer species grown in home and redcedar soils revealed many significant differences (Table S1). *A. gerardi* height in live home soils showed a strong negative feedback when compared to height in sterile home soils (*t* = 17.2, *p* < .001). Height of *A. gerardi* in sterile home soils was greater than in sterile redcedar soils (*t* = 3.3, *p* = .029), but greater than height in home live soils (*t* = 15.0, *p* < .001; Figure 2a). Height of *A. gerardi* in live home soils was significantly shorter than in live redcedar soils (*t* = −16.3, *p* < .001). Similarly, *S. scoparium* height in home sterile soils was much greater than in home live soil (*t* = 10.3, *p* < .001), indicating a strong negative feedback. *S. scoparium* height in live (*t* = 7.6, *p* < .001) and sterile (*t* = −7.7, *p* < .001) redcedar soils were shorter than in home sterile soils. There was no detectable difference in *S. scoparium* height when comparing growth in home live soils and sterile or live redcedar soils (Figure 2b). There was no detectable difference in *B. inermis* height in live home soils and sterile home soils (*t* = −2.7, *p* = .194). The height of *B. inermis* was suppressed in sterile redcedar soils relative to live (*t* = −15.2, *p* < .001) and sterile (*t* = 13.0, *p* < .001) home soils and was also suppressed in live redcedar soils relative to live (*t* = 13.5, *p* < .001) and sterile (*t* = 11.2, *p* < .001) home soils (Figure 2c). The height of *P. smithii* showed no detectable difference between live home soils and sterile home soils (*t* = 0.5, *p* = 1.0). The height of *P. smithii* growth was suppressed in sterile redcedar soils relative to sterile (*t* = −10.4, *p* < .001) and live (*t* = −13.0, *p* < .001) home soils. The height of *P. smithii* growth was also suppressed in live redcedar soils relative to sterile (*t* = 13.9, *p* < .001) and live (*t* = −16.6, *p* < .001) home soils. Live redcedar soils suppressed the height of *P. smithii* relative to growth in sterile redcedar soils (*t* = 4.5, *p* < .001; Figure 2d).

### 3.2 Plant biomass

There were many significant differences in the final shoot biomass of each species in the effects of the interaction between home or redcedar soil types and the main effects of whether the soil was live or sterilized (Table 2). Root biomass and total biomass results generally aligned with those of shoot biomass (see Figures S2, S3 and Tables S2, S3).

Plant–soil feedbacks where soil conditioned by redcedar suppressed shoot biomass were not detected for either C_4_ grass species in the study. *A. gerardi* shoot biomass in live home soils showed a strong negative feedback (estimate = −1.3, *p* < .001) when compared to the biomass of samples grown in sterile home soils. Shoot biomass of *A. gerardi* grown in live home soils was less than its biomass when grown in redcedar soils that were live (estimate = −1.6, *p* < .001) or sterile (estimate = −1.5, *p* < .001). No significant effects or interactions were found when modeling shoot biomass as a function of growth in home or redcedar soils and soil sterilization status.

**FIGURE 3:**
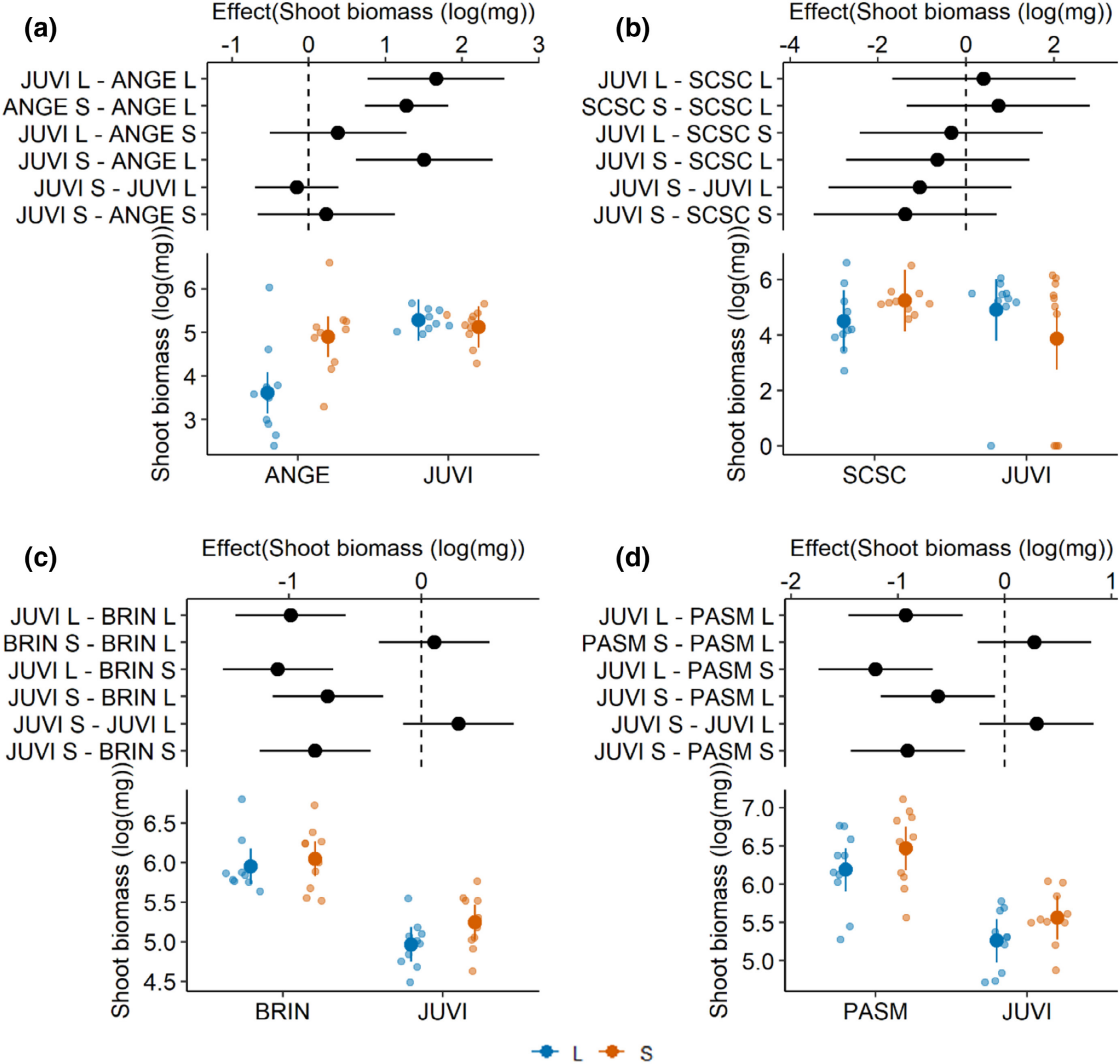
These plots illustrate the effect of home‐ and redcedar‐conditioned soils and whether the soil is sterilized (s) or live (L) on the shoot biomass of (a) *Andropogon gerardi* (ANGE), (b) *Schizachyrium scoparium* (SCSC), (c) *Bromus inermis* (BRIN), and (d) *Pascopyrum smithii* (PASM). *Juniperus virginiana* is abbreviated as JUVI. Top of each figure: Effects plot showing the difference in means between home and redcedar soils and sterilization status of those soils. The horizontal black bars show 95% confidence intervals of the effects. The vertical dashed line shows where there is no difference between groups, a 95% confidence interval that crosses this dashed line indicates no significant difference in the effects of contrasting pairs of treatment groups. The *x*‐axis scale is log_10_(biomass, mg). The *Y*‐axis lists the contrasts between each pairing of treatment types. Bottom of each figure: This portion of each plot shows the modeled response to each treatment pair, where the large solid dot is the mean and the vertical bars are the modeled 95% confidence intervals. Semi‐transparent dots illustrate the raw data for each treatment combination. Blue indicates live (L) soils and orange indicates soils that were sterilized (S).

The C_3_ grasses in this experiment showed strong negative feedbacks when grown in redcedar soil (Figure 3). However, the shoot biomass of *B. inermis* did not show any significant feedback when growth between live and sterile home soils (estimate = −0.10, *p* = .93) was contrasted. Shoot biomass of *B. inermis* was reduced when grown in live (estimate = 0.99, *p* < .001) or sterile (estimate = 0.71, *p* < .001) redcedar soils in comparison to shoot biomass in live home soils. Similarly, shoot biomass of *B. inermis* was reduced when grown in live (estimate = −1.1, *p* < .001) or sterile (estimate = 0.80, *p* < .001) redcedar soils in comparison to shoot biomass in sterile home soils. Shoot biomass of *B. inermis* did not differ when grown in live or sterile redcedar‐conditioned soils (estimate = −0.28, *p* = .29) and did not differ when grown in home live or sterile soils (estimate = −0.28, *p* = .50). Shoot biomass of *P. smithii* grown in sterile redcedar soils was reduced significantly when compared to live (estimate = 0.62, *p* = .017) or sterile (estimate = −0.91, *p* < .001) home soils and was reduced when grown in live redcedar soils when compared to live (estimate = −0.93, *p* < .001) or sterile (estimate = −1.2, *p* < .001) home soils. Shoot biomass of *P. smithii* did not differ when grown in live or sterile redcedar conditioned soils (estimate = −0.30, *p* = .44).

## 4 DISCUSSION

The growth of woody species is limited by above‐ and belowground competition during early stages of establishment in grasslands (Bush & Auken, 1990; Ward, 2020). Identifying mechanisms that could promote survivorship and growth of woody species during their seedling stage is critical to understanding how they encroach into grasslands (Van Auken, 2000). In this experiment, two of the four grass species (*Bromus inermis* and *Pascopyrum smithii*) grown in soil conditioned by redcedar experienced negative plant–soil feedback that suppressed their height and biomass. This suggests plant–soil feedback may increase survivorship of redcedar seedlings in their encroaching range depending on the local plant community at the site of establishment, much as it has done for other species combinations (Aldorfová et al., 2020).

In our experiment, grass growth in live and sterilized redcedar soil was reduced when compared to growth in live and sterilized home soils for the C_3_ grasses *B. inermis* and *P. smithii*. Plants frequently experience strong negative feedback when growing in live home soils due to accumulation of specialized pathogens (Bever, 1994; Lekberg et al., 2018; Petermann et al., 2008). Therefore, the observed suppression of grass growth in redcedar‐conditioned soils relative to home soils is noteworthy and may represent a key factor in redcedar expansion into grasslands. Negative feedbacks from dissimilar heterospecific species on target species can be derived from either an antimicrobial effect of soil biota in the conditioned soil (Haichar et al., 2014) or from the production of allelochemicals that negatively affect the growth of the target plant directly or by inhibiting the establishment of beneficial soil microbial communities (Bennett & Klironomos, 2019; Mommer et al., 2008). In this experiment, we observed the inhibition of C_3_ grass height and biomass in sterilized redcedar soils, which may be indicative that redcedar exudes an allelochemical into its near‐soil environment. In addition, our treatments had a relatively small inoculation of conditioned soil to sterilized sand (1:4), making the observation of measurable feedbacks derived from biotic and abiotic sources noteworthy.

We are uncertain why C_3_ species showed negative feedbacks and not C_4_ species. A possible explanation is that the C_3_ redcedar has novel weapons against these two species (Callaway & Ridenour, 2004; Orians & Ward, 2010). The Eurasian origins of *B. inermis* that now occupies the entire contiguous United States and the recent switch to dominance of *P. smithii* in parts of the Great Plains during the Dust Bowl could indicate that these species have had relatively limited exposure to any secondary chemicals produced by redcedar (Knapp et al., 2020; Weaver, 1942). Another possibility is that because redcedar is a C_3_ plant, it produces a stronger negative feedback with other C_3_ plants. Further study of more C_3_ grass species will be needed to determine if this is a causal relationship or a coincidence. Grasses show large variability when grown in the soil of other grasses but tend to have negative feedbacks when grown in the soil of other functional groups (Forero et al., 2022). The differential response of C_3_ and C_4_ grasses in this experiment may reflect differences in their reliance on mycorrhizal associations. Cool‐season grasses are less likely to associate with mycorrhiza, which tends to make them more self‐sufficient, whereas mycorrhizal associations are more important for warm‐season grasses (Hetrick et al., 2011). Fungal associations in C_4_ species may buffer them against the effects of allelochemicals exuded by redcedars.

The modification of the soil environment by allelopathic woody plants is an important process that can create a positive feedback for their encroachment (Caracciolo et al., 2016; Eldridge et al., 2011). Researchers have explored the possibility of allelopathy in several North American *Juniperus* species with mixed results (Norman & Anderson, 2003; Schott & Pieper, 1985). Past investigations of redcedar allelopathy have focused on germination rates of prairie plants. For example, Corbett and Lashley (2017) found redcedar litter additions did not negatively affect germination of test species. However, Stipe and Bragg (1989) noted suppression of germination for a different pool of test species grown in soil collected from a redcedar stand. Our findings take this research one step further by demonstrating the suppression of plant performance following successful germination. Taken together, the ability of redcedar to reduce the germination rate of grasses and suppress their growth following establishment may be a key factor in its successful encroachment of prairies. A future study that examines the effect of redcedar soils on grasses from their germination stage through flowering could give further insight into the overall effects of redcedar‐mediated feedbacks.

Our experimental results show a negative feedback for certain grasses grown in soil conditioned by redcedar, but interpretation of these results must also consider the myriad factors that influence plant–plant interactions in the field. Our study examined growth of individuals in a greenhouse, using potting mix and sand as soil substrates, and comparing live inoculations of conditioned soil with those that had been sterilized under heat and pressure. The microbial community of the potting soil at the onset of the training phase represents an unknown variable, outside of the mycorrhizal fungi that the manufacturers state they add. The strength of plant–soil feedbacks measured in artificial conditions have been found to be inflated relative to those observed in field conditions (Kulmatiski & Kardol, 2008). Confounding factors that could change the relative strength of feedback in field conditions include the near‐neighborhood community composition and competitive interactions. For example, we observed strong suppression of individuals of *B. inermis* and *P. smithii* grown in live and sterilized redcedar soils. In field conditions, individuals of *B. inermis* and *P. smithii* could be expected to grow in patches where they have many conspecific neighbors (Fink & Wilson, 2011; Ott & Hartnett, 2015). In the prairies of the Great Plains, *B. inermis* has been shown to have positive conspecific plant–soil feedback that can exclude heterospecific plants (Vinton & Goergen, 2006). Additionally, when *B. inermis* occurs at high density, it has been shown to be a strong competitor with redcedar seedlings (Hamati et al., 2021). In mixed‐grass prairies, *P. smithii* invests heavily in spreading its resources through rhizomes that aid in ensuring plant survival in changing conditions (Ott & Hartnett, 2015). Taken in this context, it is unlikely that the allelopathic effect of redcedar seedlings could fully displace *B. inermis* or *P. smithii* in a dense monoculture. However, if the suppressive effect of redcedar is sufficiently large to allow redcedar individuals to establish and survive long enough to overtop their competitors, then plant–soil feedbacks could be an important factor in the spread of the redcedars. Inherently, this effect will only apply to near neighbors that overlap in the rooting zone of redcedars (i.e., over a short distance). Further studies are needed to determine the strength of this effect in field conditions, the size of the area of impact around trees, the longevity of the effect in the soil, and how the strength of suppression changes with tree size or age and with the age of surrounding perennial grasses.

## AUTHOR CONTRIBUTIONS

**Leland Dwyth Bennion:** Conceptualization (lead); data curation (lead); formal analysis (lead); investigation (lead); methodology (lead); project administration (lead); resources (supporting); software (lead); supervision (supporting); validation (lead); visualization (lead); writing – original draft (lead); writing – review and editing (equal). **David Ward:** Conceptualization (supporting); data curation (supporting); formal analysis (supporting); funding acquisition (lead); investigation (supporting); methodology (supporting); project administration (equal); resources (lead); software (supporting); supervision (equal); validation (supporting); visualization (supporting); writing – original draft (supporting); writing – review and editing (equal).

## CONFLICT OF INTEREST

None declared.

### OPEN RESEARCH BADGES

This article has earned Open Data and Open Materials badges. Data and materials are available at https://doi.org/10.21038/benn.2022.0901.

## Supporting information


Appendix S1

## Data Availability

The data collected in this experiment are archived in the Open Access Kent State (OAKS) repository (https://doi.org/10.21038/benn.2022.0901).
