# Supplementary material for: Plant–soil feedback from eastern redcedar (Juniperus virginiana) inhibits the growth of grasses in encroaching range
Source: Ecol Evol. 2022 Oct 17;12(10):e9400. doi: 10.1002/ece3.9400 (PMC9596334; doi:10.1002/ece3.9400)
Supplement: Supplementary file 1 — Appendix S1 [file ECE3-12-e9400-s001.docx]

**Appendix**

**Table S1.**

This table shows contrasts between modeled height of the four study grasses grown in home and redcedar soils. The species are abbreviated using the following notation: *Andropogon gerardi* (ANGE), *Schizachyrium scoparium* (SCSC), *Bromus inermis* (BRIN), *Pascopyrum smithii* (PASM), and *Juniperus virginiana* (JUVI). These contrasts are derived from generalized additive models of log_10_(height) as a function of the interaction between the factors *soil sterilization status* (Live = L and Sterile = S) and *parent soil type* and the smoothing variables *days of growth*, *days of growth while accounting for interactions of treatment factors*, and the random intercept of *pot ID* for each plant. The significance of contrasts was calculated by comparing the ratio between the estimated marginal means of different groups of interest. If the ratio is <1, this indicates the first term of the contrast is less than the second term. Conversely, if the ratio is >1, this indicates that the first term of the contrast is greater than the second term. The significance of these differences was calculated using a Tukey adjustment of α for a family of ten estimates.

| Phytometer | Contrast | ratio | SE | df | lower CL | upper CL | null | t ratio | p |
| --- | --- | --- | --- | --- | --- | --- | --- | --- | --- |
| ANGE | ANGE S / JUVI S | 1.12 | 0.04 | 1698.20 | 1.01 | 1.25 | 1 | 3.3 | 0.029 |
| ANGE | ANGE S / ANGE L | 1.88 | 0.07 | 1698.20 | 1.68 | 2.11 | 1 | 17.2 | <0.001 |
| ANGE | ANGE S / JUVI L | 1.02 | 0.04 | 1698.20 | 0.91 | 1.15 | 1 | 0.6 | 1.000 |
| ANGE | JUVI S / ANGE L | 1.68 | 0.06 | 1698.20 | 1.50 | 1.87 | 1 | 15.0 | <0.001 |
| ANGE | JUVI S / JUVI L | 0.91 | 0.03 | 1698.20 | 0.82 | 1.02 | 1 | -2.6 | 0.207 |
| ANGE | ANGE L / JUVI L | 0.54 | 0.02 | 1698.20 | 0.48 | 0.61 | 1 | -16.3 | <0.001 |
| SCSC | JUVI S / SCSC S | 0.74 | 0.03 | 1547.97 | 0.66 | 0.84 | 1 | -7.7 | <0.001 |
| SCSC | JUVI S / JUVI L | 1.04 | 0.05 | 1547.97 | 0.90 | 1.19 | 1 | 0.8 | 0.998 |
| SCSC | JUVI S / SCSC L | 1.06 | 0.04 | 1547.97 | 0.95 | 1.18 | 1 | 1.8 | 0.761 |
| SCSC | SCSC S / JUVI L | 1.40 | 0.06 | 1547.97 | 1.22 | 1.61 | 1 | 7.6 | <0.001 |
| SCSC | SCSC S / SCSC L | 1.43 | 0.05 | 1547.97 | 1.28 | 1.60 | 1 | 10.3 | <0.001 |
| SCSC | JUVI L / SCSC L | 1.02 | 0.04 | 1547.97 | 0.90 | 1.17 | 1 | 0.6 | 1.000 |
| BRIN | BRIN S / JUVI S | 1.65 | 0.06 | 1692.73 | 1.46 | 1.87 | 1 | 13.0 | <0.001 |
| BRIN | BRIN S / BRIN L | 0.91 | 0.03 | 1692.73 | 0.82 | 1.02 | 1 | -2.7 | 0.194 |
| BRIN | BRIN S / JUVI L | 1.52 | 0.06 | 1692.73 | 1.35 | 1.71 | 1 | 11.2 | <0.001 |
| BRIN | JUVI S / BRIN L | 0.55 | 0.02 | 1692.73 | 0.49 | 0.63 | 1 | -15.2 | <0.001 |
| BRIN | JUVI S / JUVI L | 0.92 | 0.04 | 1692.73 | 0.81 | 1.05 | 1 | -2.0 | 0.578 |
| BRIN | BRIN L / JUVI L | 1.66 | 0.06 | 1692.73 | 1.48 | 1.87 | 1 | 13.5 | <0.001 |
| PASM | JUVI S / PASM S | 0.67 | 0.03 | 1704.72 | 0.60 | 0.76 | 1 | -10.4 | <0.001 |
| PASM | JUVI S / JUVI L | 1.19 | 0.05 | 1704.72 | 1.05 | 1.34 | 1 | 4.5 | <0.001 |
| PASM | JUVI S / PASM L | 0.68 | 0.02 | 1704.72 | 0.62 | 0.75 | 1 | -13.0 | <0.001 |
| PASM | PASM S / JUVI L | 1.76 | 0.07 | 1704.72 | 1.55 | 2.01 | 1 | 13.9 | <0.001 |
| PASM | PASM S / PASM L | 1.02 | 0.03 | 1704.72 | 0.92 | 1.13 | 1 | 0.5 | 1.000 |
| PASM | JUVI L / PASM L | 0.58 | 0.02 | 1704.72 | 0.52 | 0.64 | 1 | -16.6 | <0.001 |

**Table S2.**

The mean estimate, variance, and confidence intervals of effects on root biomass for contrasting interactions of each home and redcedar and soil sterilization status. Phytometers and parent soil types are abbreviated as follows: *Andropogon gerardi* (ANGE), *Schizachyrium scoparium* (SCSC), *Bromus inermis* (BRIN), *Pascopyrum smithii* (PASM), and *Juniperus virginiana* (JUVI).. Soils are either live (L) or sterile (S).

| **Phytometer** | **Contrasts** | **estimate** | **SE** | **df** | **lower CL** | **upper CL** | **t ratio** | **p** |
| --- | --- | --- | --- | --- | --- | --- | --- | --- |
| ANGE | ANGE L - JUVI L | -1.813 | 0.323 | 29.568 | -2.691 | -0.935 | -5.617 | <0.001 |
|  | ANGE L - ANGE S | -1.464 | 0.236 | 18 | -2.131 | -0.798 | -6.211 | <0.001 |
|  | ANGE L - JUVI S | -1.561 | 0.323 | 29.568 | -2.44 | -0.683 | -4.837 | <0.001 |
|  | JUVI L - ANGE S | 0.349 | 0.323 | 29.568 | -0.53 | 1.227 | 1.08 | 0.704 |
|  | JUVI L - JUVI S | 0.252 | 0.236 | 18 | -0.415 | 0.918 | 1.067 | 0.713 |
|  | ANGE S - JUVI S | -0.097 | 0.323 | 29.568 | -0.975 | 0.781 | -0.3 | 0.99 |
| SCSC | JUVI L - SCSC L | 0.381 | 0.802 | 36 | -1.778 | 2.541 | 0.476 | 0.964 |
|  | JUVI L - JUVI S | 0.968 | 0.802 | 36 | -1.191 | 3.127 | 1.207 | 0.626 |
|  | JUVI L - SCSC S | -0.498 | 0.802 | 36 | -2.658 | 1.661 | -0.621 | 0.925 |
|  | SCSC L - JUVI S | 0.587 | 0.802 | 36 | -1.573 | 2.746 | 0.732 | 0.884 |
|  | SCSC L - SCSC S | -0.88 | 0.802 | 36 | -3.039 | 1.28 | -1.097 | 0.694 |
|  | JUVI S - SCSC S | -1.466 | 0.802 | 36 | -3.626 | 0.693 | -1.829 | 0.277 |
| BRIN | BRIN L - JUVI L | 1.162 | 0.236 | 35.948 | 0.525 | 1.799 | 4.914 | <0.001 |
|  | BRIN L - BRIN S | -0.169 | 0.232 | 18 | -0.824 | 0.487 | -0.728 | 0.885 |
|  | BRIN L - JUVI S | 0.357 | 0.236 | 35.948 | -0.279 | 0.994 | 1.512 | 0.441 |
|  | JUVI L - BRIN S | -1.331 | 0.236 | 35.948 | -1.968 | -0.694 | -5.628 | <0.001 |
|  | JUVI L - JUVI S | -0.805 | 0.232 | 18 | -1.46 | -0.149 | -3.469 | 0.013 |
|  | BRIN S - JUVI S | 0.526 | 0.236 | 35.948 | -0.111 | 1.163 | 2.226 | 0.136 |
| PASM | JUVI L - PASM L | -1.185 | 0.17 | 18 | -1.665 | -0.706 | -6.984 | <0.001 |
|  | JUVI L - JUVI S | -0.538 | 0.154 | 19 | -0.97 | -0.106 | -3.503 | 0.012 |
|  | JUVI L - PASM S | -1.723 | 0.229 | 36.42 | -2.339 | -1.107 | -7.529 | <0.001 |
|  | PASM L - JUVI S | 0.647 | 0.229 | 36.42 | 0.031 | 1.263 | 2.827 | 0.036 |
|  | PASM L - PASM S | -0.538 | 0.154 | 19 | -0.97 | -0.106 | -3.503 | 0.012 |
|  | JUVI S - PASM S | -1.185 | 0.17 | 18 | -1.665 | -0.706 | -6.984 | <0.001 |

**Table S3.**

The mean estimate, variance, and confidence intervals of effects on total biomass for contrasting interactions of each home and redcedar (JUVI) and soil sterilization status. Phytometers and conditioned soil types are abbreviated as follows: *Andropogon gerardi* (ANGE), *Bromus inermis* (BRIN), *Pascopyrum smithii* (PASM), *Schizachyrium scoparium* (SCSC), and *Juniperus virginiana* (JUVI). Soils are either live (L) or sterile (S). Non-integers for d.f. derive from mixed models. See details in Figure S3.

| **Phytometer** | **Contrast** | **estimate** | **SE** | **df** | **lower CL** | **upper CL** | **t.ratio** | **p** |
| --- | --- | --- | --- | --- | --- | --- | --- | --- |
| ANGE | ANGE L - JUVI L | -1.764 | 0.318 | 28.088 | -2.631 | -0.896 | -5.551 | <0.001 |
|  | ANGE L - ANGE S | -1.408 | 0.218 | 18 | -2.023 | -0.793 | -6.468 | <0.001 |
|  | ANGE L - JUVI S | -1.537 | 0.318 | 28.088 | -2.404 | -0.67 | -4.837 | <0.001 |
|  | JUVI L - ANGE S | 0.356 | 0.318 | 28.088 | -0.511 | 1.223 | 1.12 | 0.68 |
|  | JUVI L - JUVI S | 0.227 | 0.218 | 18 | -0.388 | 0.842 | 1.042 | 0.728 |
|  | ANGE S - JUVI S | -0.129 | 0.318 | 28.088 | -0.996 | 0.738 | -0.406 | 0.977 |
| BRIN | BRIN L - JUVI L | 1.14 | 0.201 | 36 | 0.599 | 1.68 | 5.678 | <0.001 |
|  | BRIN L - BRIN S | -0.13 | 0.201 | 36 | -0.671 | 0.411 | -0.647 | 0.916 |
|  | BRIN L - JUVI S | 0.451 | 0.201 | 36 | -0.089 | 0.992 | 2.248 | 0.13 |
|  | JUVI L - BRIN S | -1.27 | 0.201 | 36 | -1.81 | -0.729 | -6.325 | <0.001 |
|  | JUVI L - JUVI S | -0.688 | 0.201 | 36 | -1.229 | -0.148 | -3.429 | 0.008 |
|  | BRIN S - JUVI S | 0.581 | 0.201 | 36 | 0.041 | 1.122 | 2.896 | 0.031 |
| PASM | JUVI L - PASM L | -1.079 | 0.153 | 18 | -1.511 | -0.647 | -7.062 | <0.001 |
|  | JUVI L - JUVI S | -0.435 | 0.143 | 19 | -0.838 | -0.032 | -3.032 | 0.032 |
|  | JUVI L - PASM S | -1.514 | 0.21 | 36.703 | -2.078 | -0.95 | -7.223 | <0.001 |
|  | PASM L - JUVI S | 0.644 | 0.21 | 36.703 | 0.08 | 1.208 | 3.073 | 0.02 |
|  | PASM L - PASM S | -0.435 | 0.143 | 19 | -0.838 | -0.032 | -3.032 | 0.032 |
|  | JUVI S - PASM S | -1.079 | 0.153 | 18 | -1.511 | -0.647 | -7.062 | <0.001 |
| SCSC | JUVI L - SCSC L | 0.325 | 0.868 | 36 | -2.012 | 2.662 | 0.375 | 0.982 |
|  | JUVI L - JUVI S | 1.141 | 0.868 | 36 | -1.196 | 3.479 | 1.315 | 0.56 |
|  | JUVI L - SCSC S | -0.488 | 0.868 | 36 | -2.826 | 1.849 | -0.563 | 0.942 |
|  | SCSC L - JUVI S | 0.816 | 0.868 | 36 | -1.521 | 3.154 | 0.94 | 0.783 |
|  | SCSC L - SCSC S | -0.814 | 0.868 | 36 | -3.151 | 1.524 | -0.937 | 0.785 |
|  | JUVI S - SCSC S | -1.63 | 0.868 | 36 | -3.967 | 0.708 | -1.878 | 0.255 |


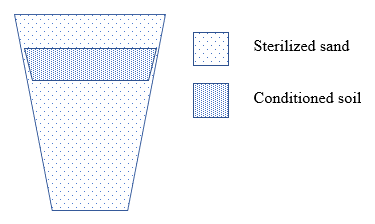


**Figure S1:** This is a basic illustration of how substrates were combined in 2.8 L pots. Sand was sterilized in an autoclave and cooled prior to being added to each pot. Conditioned soils from the training phase were added and then capped with additional sand. One individual phytometer was transplanted into each prepared pot.


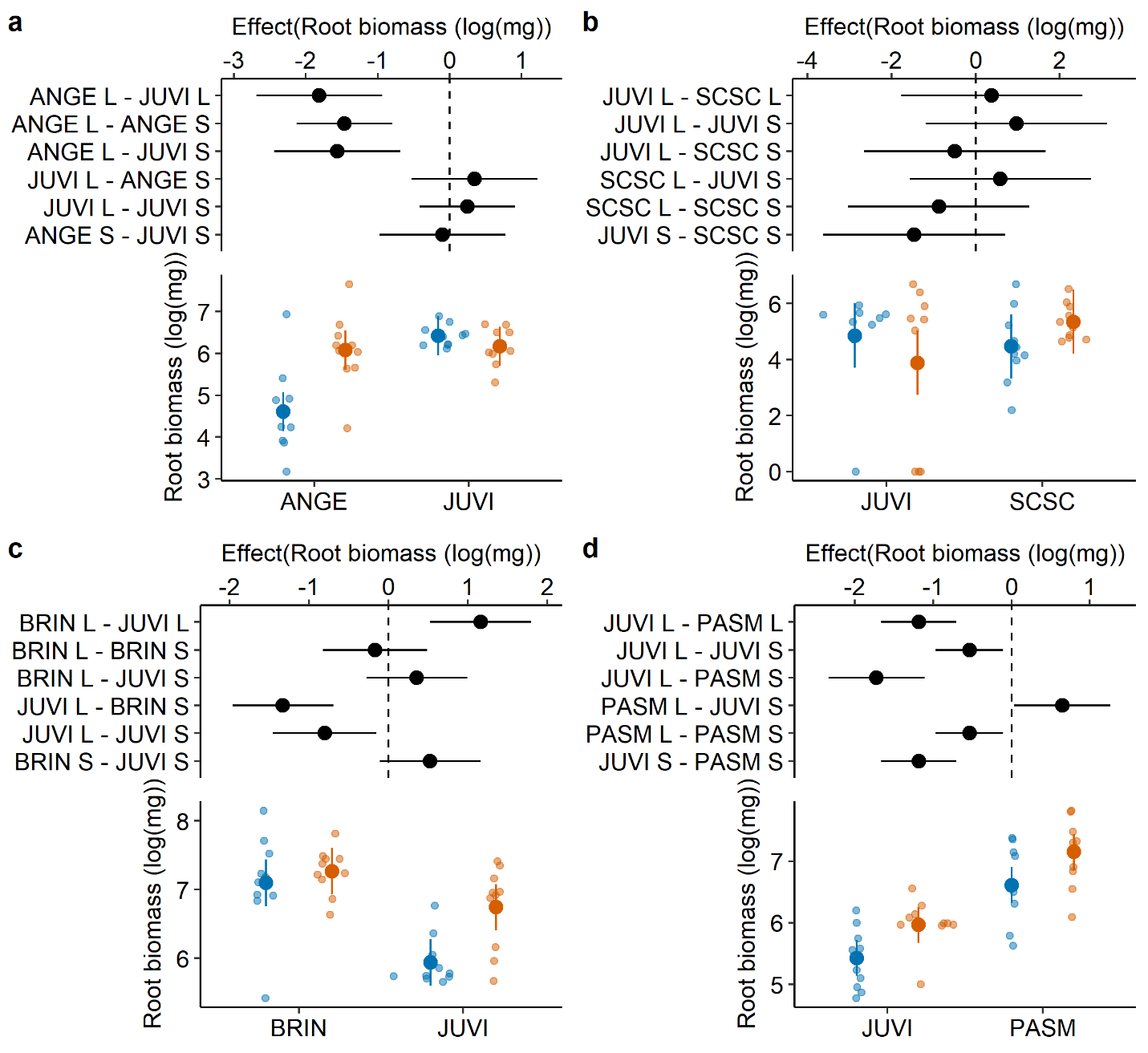


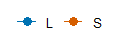


**Figure S2.** These plots illustrate the effects of home- and redcedar-conditioned soils and whether the soil is sterilized (S) or live (L) on the root biomass of (a) *Andropogon gerardi* (ANGE), (b) *Schizachyrium scoparium* (SCSC), (c) *Bromus inermis* (BRIN) and (d) *Pascopyrum smithii* (PASM). *Juniperus virginiana* is abbreviated as JUVI. Top of each figure: Effects plot showing the difference in means between home and redcedar soils and sterilization status of those soils. The horizontal black bars show 95% confidence intervals of the effects. The vertical dashed line shows where there is no difference between groups, a 95% confidence interval that crosses this dashed line indicates no significant difference in the effects of contrasting pairs of treatment groups. The x-axis scale is log_10_(biomass, mg). The Y-axis lists the contrasts between each pairing of treatment types. Bottom of each figure: This portion of each plot shows the modeled response to each treatment pair, where the large solid dot is the mean and the vertical bars are the modeled 95% confidence intervals. Dots illustrate the raw data for each treatment combination. Blue coloration indicates live (L) soils and orange coloration indicates soils that were sterilized (S).


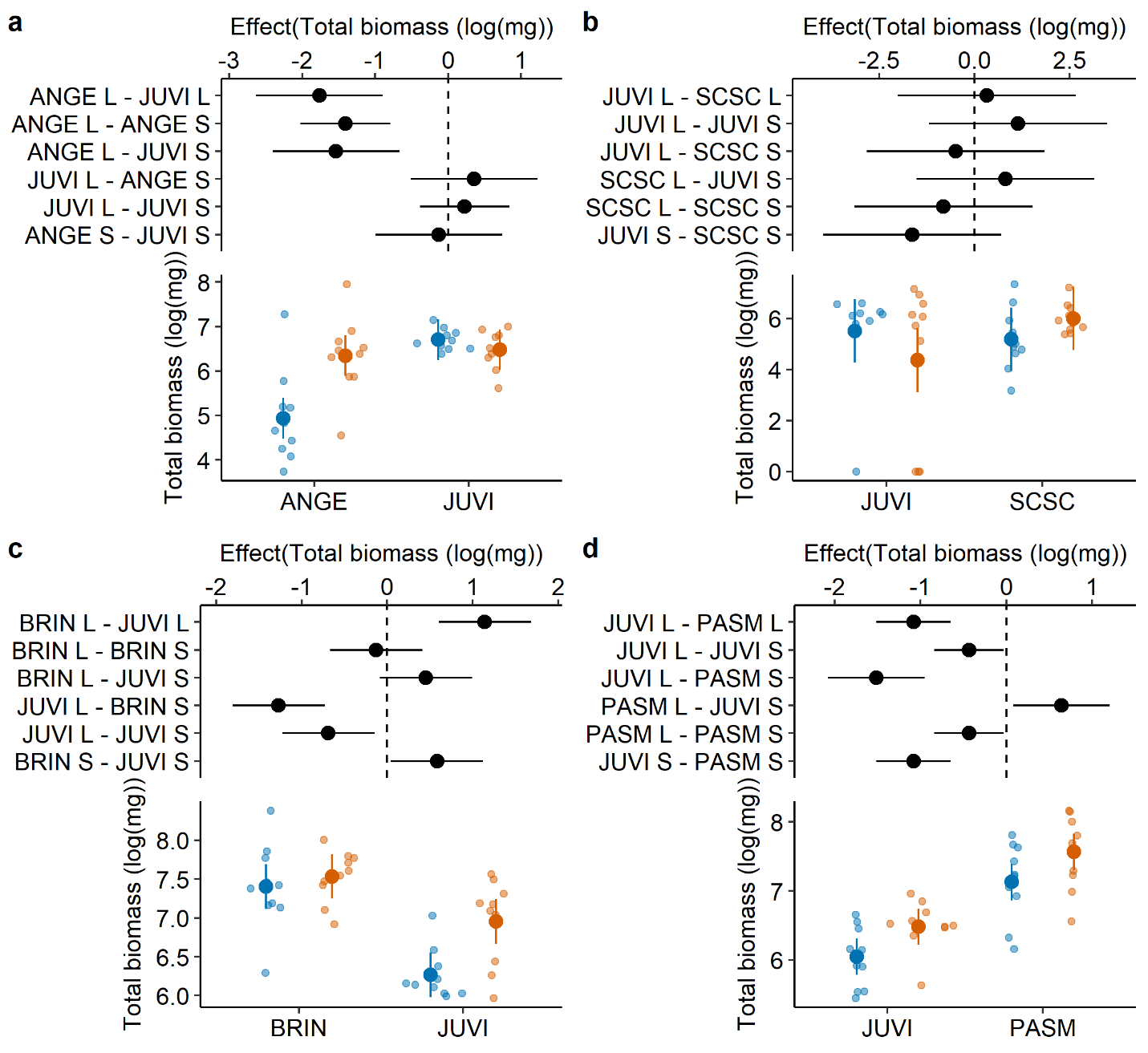


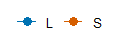


**Figure S3.** These plots illustrate the effect of home- and redcedar-conditioned soils and whether the soil is sterilized (S) or live (L) on the total biomass of (a) *Andropogon gerardi* (ANGE), (b) *Schizachyrium scoparium* (SCSC), (c) *Bromus inermis* (BRIN), and (d) *Pascopyrum smithii* (PASM). *Juniperus virginiana* is abbreviated as JUVI. Top of each figure: Effects plot showing the difference in means between home and redcedar soils and sterilization status of those soils. The horizontal black bars show 95% confidence intervals of the effects. The vertical dashed line shows where there is no difference between groups, a 95% confidence interval that crosses this dashed line indicates no significant difference in the effects of contrasting pairs of treatment groups. The x-axis scale is log_10_(biomass, mg). The Y-axis lists the contrasts between each pairing of treatment types. Bottom of each figure: This portion of each plot shows the modeled response to each treatment pair, where the large solid dot is the mean and the vertical bars are the modeled 95% confidence intervals. Dots illustrate the raw data for each treatment combination. Blue indicates live (L) soils and orange indicates soils that were sterilized (S).
